# Electrocardiographic RR Interval Dynamic Analysis to Identify Acute Stroke Patients at High Risk for Atrial Fibrillation Episodes During Stroke Unit Admission

**DOI:** 10.1007/s12975-018-0645-8

**Published:** 2018-07-03

**Authors:** Alessandro Adami, Carolina Gentile, Thomas Hepp, Giulio Molon, Gian Luigi Gigli, Mariarosaria Valente, Vincent Thijs

**Affiliations:** 1 Stroke Center, Ospedale Sacro Cuore–Don Calabria, Negrar, Verona Italy; 2 Neurology Clinic, University of Udine, Udine, Italy; 3 Apoplex Medical Technologies GmbH, Pirmasens, Germany; 4 Cardiology Department, Ospedale Sacro Cuore–Don Calabria, Negrar, Verona Italy; 5 Stroke Division, Florey Institute of Neuroscience and Mental Health, University of Melbourne, Heidelberg, Victoria Australia

**Keywords:** Atrial fibrillation, Stroke, Heart rate variability, Continuous cardiac monitoring

## Abstract

Patients at short-term risk of paroxysmal atrial fibrillation (PAF) often exhibit increased RR interval variability during sinus rhythm. We studied if RR dynamic analysis, applied in the first hours after stroke unit (SU) admission, identified acute ischemic stroke patients at higher risk for subsequent PAF episodes detected within the SU hospitalization. Acute ischemic stroke patients underwent continuous cardiac monitoring (CCM) using standard bedside monitors immediately after SU admission. The CCM tracks from the first 48 h were analyzed using a telemedicine service (SRA clinic, Apoplex Medical, Germany). Based on the RR dynamics, the stroke risk analysis (SRA) algorithm stratified the risk for PAF as follows: low risk for PAF, high risk for PAF, presence of manifest AF. The subsequent presence/absence of PAF during the whole SU hospitalization was ruled out using all available CCMs, standard ECGs, or 24-h Holter ECGs. Two hundred patients (40% females, mean age 71 ± 16 years) were included. According to the initial SRA analysis, 111 patients (56%) were considered as low risk for PAF, 52 (26%) as high risk while 37 patients (18%) had manifest AF. A low-risk level SRA was associated with a reduced probability for subsequent PAF detection (1/111, 0.9%, 95% CI 0–4.3%) while a high-risk level SRA predicted an increased probability (20/52, 38.5% (95% CI 25–52%). RR dynamic analysis performed in the first hours after ischemic stroke may stratify patients into categories at low or high risk for forthcoming paroxysmal AF episodes detected within the SU hospitalization.

## Introduction

Detection of paroxysmal atrial fibrillation (PAF) after an ischemic stroke is of paramount importance since patients with AF benefit from oral anticoagulation more than from standard antiplatelet therapy [1, 2]. The diagnosis of PAF is often challenging, as it may remain undetected on conventional ECG and a Holter ECG as well as short-term continuous cardiac monitoring (CCM) provided by stroke unit (SU) monitors [3, 4]. Current guidelines recommend the use of CCM at least for the first 24–72 h after stroke [1, 2]; however, there is at present no consensus on the optimal method and duration of CCM in SU settings [5]. Moreover, conventional bedside monitors are usually withheld after the first days as they severely restrict the patient mobility and are often poor tolerated. The search for PAF in SU can be further pursued with longer term CCM using mobile telemetry devices, repeat Holters, and patch-type devices [5]. However, access to these techniques is currently limited by their economic costs, the lack of standardized approach, and organizational burden. Thus, a large number of stroke patients with undiagnosed PAF may not receive an optimal antithrombotic therapy.

The ability to ascertain those patients at higher AF risk within the first days after stroke could improve the patient selection for longer term CCM throughout the SU stay. Clinical features (such as age, sex, the CHA_2_-DS_2_-VASc score) and electrocardiographic or echocardiographic (left atrial volume, premature atrial complexes, PR interval) predictors for PAF have been assessed. However, their clinical effectiveness is unclear [6].

SRA clinic is a telemedicine service based upon RR interval dynamic analysis providing automated AF detection for SU patients [7]. The stroke risk analysis (SRA) algorithm had been demonstrated [8, 9] able to identify abnormalities suggestive of increased PAF risk in patients with a previously diagnosed PAF but at sinus rhythm during the analysis with a 60% sensitivity and 99% specificity. Therefore, the SRA algorithm may be used to identify those acute stroke patients at higher risk for forthcoming PAF episodes.

In this study, we applied SRA analysis on CCM obtained within the initial 48 h from SU admission. We hypothesized that this technique would identify patients at higher risk for PAF detected during SU hospitalization.

## Methods

Within an unsponsored investigator-initiated observational study approved by the local ethical committee, we investigated unselected acute ischemic stroke and TIA patients consecutively admitted in a single stroke center. Enrollment was retrospective from January 2014 to January 2015 and then prospective until December 2016. Inclusion criteria were as follows: stroke/TIA diagnosis confirmed by a neurologist, hospital admission within 24 h from symptom onset, availability of initial 48-h CCM, and availability of standard ECG or a 24-h Holter ECG or further CCM after the first days of monitoring. Inability to give consent and the presence of implantable rhythm control devices were exclusion criteria. For all patients, a clinical history was collected and National Institutes of Health Stroke Scale (NIHSS) admission and CHA_2_-DS_2_-Vasc scores calculated. Standard investigations comprised brain CT/MRI, echocardiography, and carotid ultrasound study. Additional ECGs, a 24-h Holter ECG, and further CCM monitoring were performed according to the clinician’s discretion. Patients treated with IV thrombolysis were admitted in the ICU department for the first 24–48 h and initiated their initial CCM when transferred into the SU.

After SU admission, CCM for consecutive 48 h was immediately initiated using standard bedside monitors (Intellivue MP40, Philips). After 48 h, CCM was withheld to permit mobilization unless further monitoring was deemed clinically necessary in patients with more severe/unstable clinical conditions. CCM ECG tracks were sent via a secure internet connection to the SRA server (Apoplex Medical Technologies, Pirmasens, Germany) for the assessment of RR interval dynamics calculated on overall 48-h recordings. The details of SRA data processing have been previously described [8, 9]. Briefly, the AF risk is associated with the presence of premature atrial complexes, atrial tachycardia, and other ectopic activities. These abnormalities alter atrioventricular nodal conduction and result in changes in the ventricular response that are often not detected by conventional linear assessment of heart rate variability. The SRA algorithm assesses the AF risk in three steps. QRS complexes are first identified on ECG to create a RR interval list. RR intervals are normalized by dividing them by the mean of the two corresponding RR intervals [(*R*_*i*_ − *R*_*i*_ + 1) ∕ (*R*_*i*_ + *R*_*i*_ + 1)]. These RR intervals are then used to calculate various, mostly nonlinear, mathematical parameters such as the following: standard deviation of the minor and major axis of the Poincaré plots and their ratio; RR fluctuation based upon different analyses of consecutive RR intervals; the number of premature atrial complexes without sinus nodal reset. Finally, entropic analysis of RR intervals regularity is applied.

The SRA algorithm classified initial 48-h CCM tracks as (1) no presence of AF and low risk for AF; (2) no AF and high risk for AF; and (3) presence of manifest episodes of AF. In the case of detected AF, the SRA service provided source ECG tracks for clinical confirmation of AF. SRA clinic detection of manifest AF episodes includes only episodes > 30 s.

In the presence of manifest AF on ECG data, SRA clinic has a 99% sensitivity and specificity compared to Holter ECG [9].

At discharge, for each patient, the heart rhythm was adjudicated by a cardiologist as sinus rhythm/PAF/AF based on all available ECGs, a Holter ECG, and CCM tracks (including the SRA algorithm provided data).

## Statistical Analysis

In univariate analysis, we compared the risk factors that were different between patients at low risk of AF, at high risk of AF, and with manifest AF, using ANOVA for continuous data (age, NIHSS, QTc interval) and the Pearson chi-square test for categorical data. Pairwise post hoc comparisons were performed with the Bonferroni correction. We subsequently compared risk factors for subsequent diagnosis of AF in the subgroup of patients without manifest AF on SRA clinic assessment. Logistic regression was then performed in the subgroup of patients without manifest AF on SRA clinic assessment with presence of AF as the dependent and SRA clinic assessment as low or high risk, previous diagnosis of PAF/AF and CHA_2_D_2_-Vasc score as the independent variables. Because of the limited number of patients with a further diagnosis of AF, we did not further adjust for other factors. The analyses were performed using SPSS 21.0 (IBM). A *p* value of 0.05 was considered significant.

## Results

Between January 2014 and December 2016, 259 patients were considered for inclusion in the study. Of these, 59 were excluded for the following reasons: hospital admission later than 24 h from symptom onset (8); lack of early CCM (23); presence of ICDs (10); refusal/inability to participate in the study (18). Two hundred patients (99 retrospective, 40% females, mean age 71 ± 16 years) with ischemic stroke (*n* = 187) and TIA (*n* = 13) were finally enrolled. Stroke/TIA etiologic TOAST classification of enrolled patients was as follows: large artery atherosclerosis 11 (5.5%) patients; lacunar 20 (10%); other determined etiology 9 (4.5%); cardioembolic 62 (31%); cryptogenic (more than one cause or incomplete evaluation) 13 (6.5%); embolic stroke of undetermined source 85 (42.5%). One hundred and fifty-six patients (77%) initiated CCM within the same day of symptom onset while 44 patients treated with I.V thrombolysis and first admitted to the ICU initiated CCM 24 to 48 h from hospital admission.

Table 1 presents a summary of baseline characteristics of the patients. There were no differences in the clinical and instrumental findings between the retrospective and prospective patients cohort. On initial CCM, AF risk was considered low in 111 (55.5%) patients and high in 52 (26%) while 37 patients (18.5%) had manifest AF. Significant differences among the three groups were found with respect to age, risk factors (hypertension, smoking, CHA_2_DS_2_-VASC score), stroke severity, thrombolysis rates, and left atrial volume. The low-risk group patients were younger compared to the high-risk (mean difference, 15.3 years, 95% CI 10–20) and the manifest AF groups (15 years, 95% CI 9–21). The manifest AF group had a higher baseline NIHSS compared to the low (*p* = 0.001) and high AF risk groups (*p* = 0.001). There was no difference in NIHSS between low and high risk AF groups. The CHA_2_-DS_2_-Vasc scores and the QTc values were higher in the high AF and manifest AF groups compared to the low-risk group (both *p* = 0.001), but there were no significant differences between the high-risk and the manifest AF groups. There were no differences in the rate of previously diagnosed PAF, while the use of class I–III–IV antiarrhythmic drugs was higher in high-risk and AF patients. The mean SU stay was 9.7 ± 4 days without differences between the three groups.

**Table 1:** Patient characteristics

Patient characteristics	Low AF risk, *N* = 111	High AF risk, *N* = 52	Manifest AF, *N* = 37	*p*
Age (years)	65 ± 14	80 ± 8	80 ± 12	< 0.001
Sex, female	39 (35)	22 (42)	19 (51)	0.202
Current smoking	20 (18)	1 (2)	3 (8)	0.009
Hypertension	70 (63)	42 (81)	33 (89)	0.003
Diabetes	15 (13)	12 (23)	8 (22)	0.249
Dyslipidemia	44 (40)	17 (33)	10 (27)	0.338
Previous AF/PAF diagnosis	6 (5)	7 (13)	5 (13)	0.140
Antiarrhythmic drugs	24 (22)	22 (42)	18 (50)	0.001
CHA2-DS2-Vasc				
Median	4.5	5	6	
25th percentile	3	5	5	< 0.001
75th percentile	6	6	6	
NIHSS	4 ± 5	5 ± 6	11 ± 8	0.009
QTc (ms)	430 ± 34	448 ± 29	462 ± 33	< 0.001
Thrombolysis	17 (15)	6 (12)	12 (33)	0.023
Left atrial volume				
Normal (< 40 mL/m^2^)	65 (59)	21 (40)	4 (11)	
Enlarged (40–45 mL/m^2^)	11 (10)	7 (13)	4 (11)	< 0.001
Severely enlarged (> 45 mL/m^2^)	8 (7)	13 (25)	15 (41)	
Not available	27 (24)	11 (21)	14 (38)	
Left atrial diameter				
Normal (< 39 mm)	33 (30)	12 (29)	3 (8)	0.095
Enlarged (39–50 mm)	40 (36)	19 (45)	15 (41)	
Severely enlarged (> 50 mm)	5 (5)	5 (12)	4 (11)	
Not available	33 (30)	6 (14)	15 (41)	
Final heart rhythm				
Sinus rhythm	110 (99)	32 (61.5)	0	
PAF	1 (1)	20 (38.5)	12 (32)	< 0.001
Permanent/persistent AF	0	0	25 (68)	

During SU stay, AF was evident in 58 (29%) patients, in 33 (16.5%) as paroxysmal, and in 25 (12.5%) as permanent/persistent. AF was detected in 1/110 low-risk patients (0.9%; 95% CI 0–4.9%) whereas 20/52 (38.5%; 95% CI 25–52%) of high-risk patients had a diagnosis of AF. In the detection of AF, SRA exhibited 100% sensitivity and 97% specificity compared to CCM. Those six patients with AF on SRA not confirmed on source CCM were classified as high-risk for AF.

In univariate analysis, the presence of PAF among patients categorized as low- or high-risk of AF was associated with SRA category, age, previous PAF/AF history, use of antiarrhythmic drugs, CHA2-DS2-Vasc, and QTc duration (Table 2). In an exploratory multivariate model, after correction for CHA_2_DS_2_-VASC score and previous AF/PAF diagnosis, SRA risk score remained a significant predictor for a final diagnosis of AF (Table 3).

**Table 2:** Comparison between patients with and without paroxysmal atrial fibrillation (PAF)

Patient characteristics	PAF, *N* = 21	No AF, *N* = 142	*p*
Age (years)	81 ± 9	67 ± 15	<0.001
Sex, female	8 (38)	53 (37)	0.946
Current smoking	1 (5)	20 (14)	0.234
Hypertension	17 (81)	95 (67)	0.195
Diabetes	3 (14)	24 (17)	0.763
Dyslipidemia	7 (33)	54 (38)	0.678
Previous AF/PAF diagnosis	5(24)	8 (6)	0.004
Antiarrhythmic drugs	11 (52)	35(25)	0.009
CHA_2_DS_2_-Vasc			
Median	5	4.5	
25th percentile	4	3	0.013
75th percentile	6	6	
NIHSS	7 ± 7	4 ± 5	0.146
QTc (ms)	458 ± 30	432 ± 34	0.002
Thrombolysis	3 (15)	20 (14)	0.922
Left atrial volume			
Normal (< 40 mL/m^2^)	9 (50)	77 (72)	
Enlarged (40–45 mL/m^2^)	4 (22)	14 (13)	0.175
Severely enlarged (> 45 mL/m^2^)	5 (28)	16 (15)	
Not available	3	35	
Left atrial diameter			
Normal (< 39 mm)	2 (18)	43 (41)	0.296
Enlarged (39–50 mm)	8 (73)	51 (50)	
Severely enlarged (> 50 mm)	1(9)	9 (9)	
Not available	10	39	
SRA risk score			
High-risk score	20 (95.2%)	32 (22.5%)	< 0.001

**Table 3:** Multivariate analysis

Patient characteristics	Hazard Ratio	Lower 95% CI	Higher 95% CI	*p*
Previous AF/PAF diagnosis	4.27	0.84	21.7	0.080
CHA_2_DS_2_-VASC score	0.96	0.56	1.63	0.870
High-risk score SRA	70.1	7.8	632	0.000

## Discussion

In this study, electrocardiographic RR dynamics analysis, performed in the first 48 h from stroke unit admission, was able to stratify patients at high or low short-term risk for PAF. Patients at low risk with SRA analysis were unlikely to develop AF during the stay in the SU, whereas about 1/3 patients identified as high risk had a subsequent diagnosis of PAF. It is noteworthy that our analysis did not require additional investigations but were obtained using already available data, i.e., the CCM provided by conventional stroke unit monitors.

Our results are in accordance with the previously published studies. In our cohort, the total AF rate was 29%, while PAF was detected in 16.5% of cases. In a recent global survey [10], the AF prevalence among acute stroke patients was 28% while in two systematic reviews, the expected yield of detecting PAF in a standard SU setting was estimated to be 14.7 [1, 2]. Bettin et al. [11] applied SRA analysis to 106 patients with acute stroke of unknown etiology. These patients were followed up with an implantable loop recorder. On long-term monitoring, 13 patients with PAF were detected, 4% in the low-risk group and 33% in the high-risk group.

**Fig. 1:**
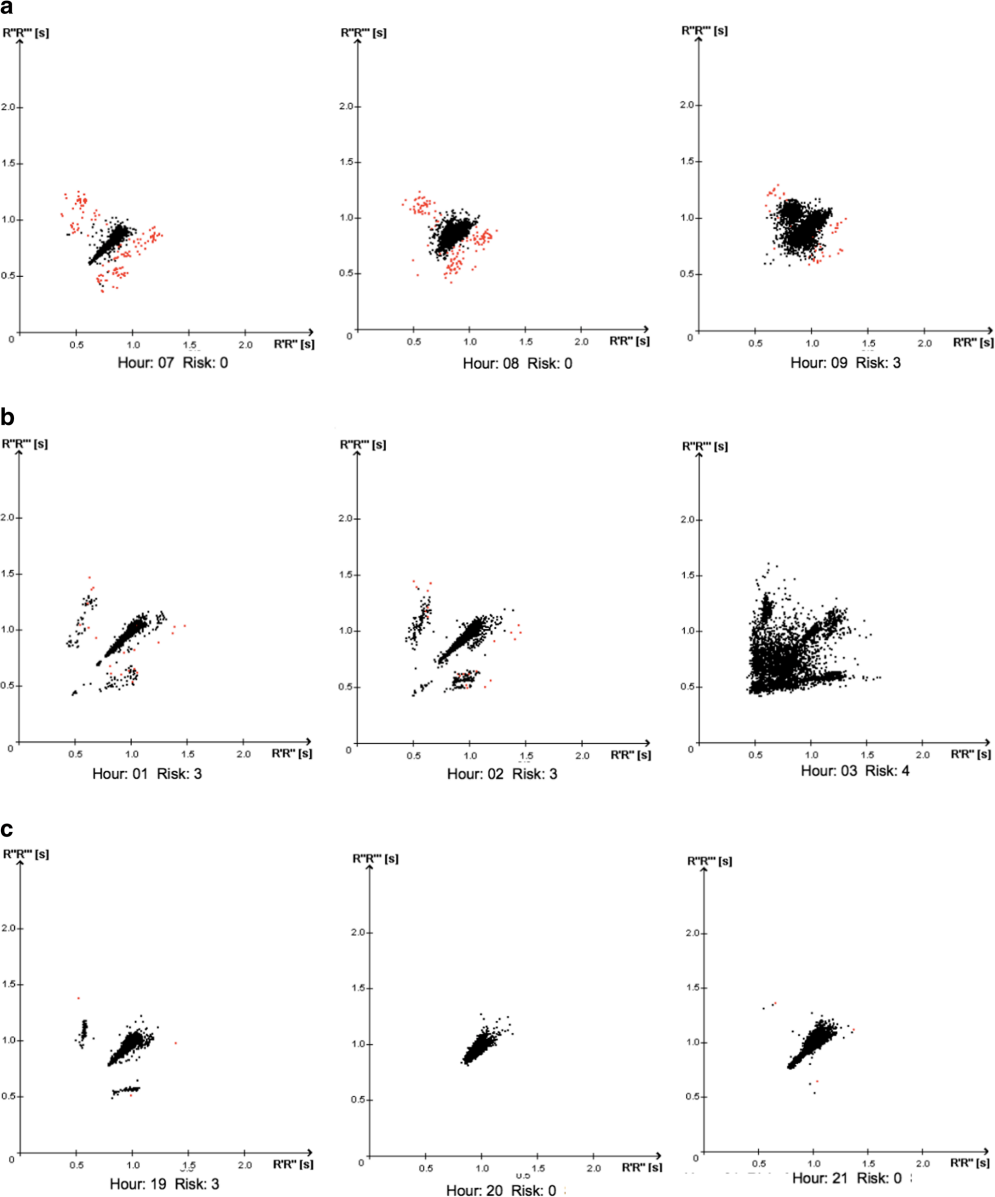
Examples of RR interval variability during continuous cardiac monitoring. Each plot represents 1 h of monitoring (the Lorentz plots: each RR interval is plotted as a function of the preceding RR interval). **a** Transitioning from low to high AF risk. **b** Transitioning from high risk to manifest AF. **c** Transitioning from high risk to low risk. AF atrial fibrillation, s seconds, Risk SRA clinic risk grade for atrial fibrillation. 0 = low risk; 3 = high risk; 4 = manifest AF (see the “Methods” section)

Rizos et al. [12] investigated 136 acute stroke patients applying SRA analysis to 1–2 h ECG recordings obtained in emergency room. The patients were then followed with CCM for ≥ 48 h after SU admittance. SRA stratified 70/136 patients as low risk and in 7/70 of these PAF was subsequently detect at CCM (this accounting for a false negative rate of 10%). In our sample, only 1/111 patient of the low-risk group developed PAF. This difference between the two studies may depend on the fact that we stratified the PAF risk analyzing with SRA 48 h of CCM rather than 1–2 h of ECG recordings. In fact, RR dynamics, as Rizos et al. [12] found, can consistently vary within contiguous hours (Fig. 1), and thus, the evaluation of longer time periods might provide a more accurate PAF risk. Interestingly, 14/34 patients classified at high risk in the study were then diagnosed with PAF, a similar percentage as in our sample (41 vs 38%).

The main limitation of this study was the lack of a predefined standard amount of ECG recordings for AF detection. After the first 48 h CCM, the use of additional ECGs, a Holter ECG, or further CCM for the detection of PAF was not standardized and left to the discretion of the treating physicians. Some patients (for example those with more severe strokes) may have received longer CCM and more ECGs/Holter ECGs than others and this may have led to increased opportunities for PAF detection. We did not report the duration of the detected PAF episodes. As pointed out by Kishore et al., in clinical acute stroke studies PAF is frequently poorly defined and there is a lack in a clear distinction between AF/PAF as well as in reporting the length of PAF episodes. The estimation of AF burden might be more important in primary than in secondary stroke prevention. Finally, our multivariate model must be considered exploratory as the relatively small sample size and several factors were associated in univariate analysis with the presence of AF.

In conclusion, using standard CCM monitoring and application of a commercial software algorithm, we have found that acute ischemic stroke patients with increased RR dynamics are at higher risk of PAF during stroke unit admission. This information could be of value to decide on longer term monitoring within the stroke unit hospitalization.
